# A Plant Model of *α*-Synucleinopathy: Expression of *α*-Synuclein A53T Variant in Hairy Root Cultures Leads to Proteostatic Stress and Dysregulation of Iron Metabolism

**DOI:** 10.3390/applbiosci3020016

**Published:** 2024-05-20

**Authors:** Jasmina Kurepa, Kristen A. Bruce, Greg A. Gerhardt, Jan A. Smalle

**Affiliations:** 1 Department of Plant and Soil Sciences, Martin-Gatton College of Agriculture Food and Environment, Kentucky Tobacco Research & Development Center, University of Kentucky, Lexington, KY 40546, USA; 2 Naprogenix, Inc.^™^, UK-AsTeCC, 145 Graham Avenue, Lexington, KY 40506, USA; 3 Brain Restoration Center, University of Kentucky, Lexington, KY 40536, USA; 4 Department of Neurosurgery, University of Kentucky, Lexington, KY 40536, USA; 5 Department of Neuroscience, University of Kentucky, Lexington, KY 40536, USA; 6 Department of Neurology, University of Kentucky, Lexington, KY 40536, USA

**Keywords:** *α*-synuclein, *Lobelia cardinalis*, *Polygonum multiflorum*, *Artemisia annua*, *Salvia miltiorrhiza*, proteostasis, iron homeostasis, synucleinopathy models

## Abstract

Synucleinopathies, typified by Parkinson’s disease (PD), entail the accumulation of *α*-synuclein (*α*Syn) aggregates in nerve cells. Various *α*Syn mutants, including the *α*Syn A53T variant linked to early-onset PD, increase the propensity for *α*Syn aggregate formation. In addition to disrupting protein homeostasis and inducing proteostatic stress, the aggregation of *α*Syn in PD is associated with an imbalance in iron metabolism, which increases the generation of reactive oxygen species and causes oxidative stress. This study explored the impact of *α*Syn A53T expression in transgenic hairy roots of four medicinal plants (*Lobelia cardinalis*, *Artemisia annua*, *Salvia miltiorrhiza*, and *Polygonum multiflorum*). In all tested plants, *α*Syn A53T expression triggered proteotoxic stress and perturbed iron homeostasis, mirroring the molecular profile observed in human and animal nerve cells. In addition to the common eukaryotic defense mechanisms against proteostatic and oxidative stresses, a plant stress response generally includes the biosynthesis of a diverse set of protective secondary metabolites. Therefore, the hairy root cultures expressing *α*Syn A53T offer a platform for identifying secondary metabolites that can ameliorate the effects of *α*Syn, thereby aiding in the development of possible PD treatments and/or treatments of synucleinopathies.

## 1. Introduction

*α*-synuclein (*α*Syn) is a soluble protein highly expressed in the central nervous system, comprising approximately 1% of neuronal cytoplasmic protein [1]. Its exact function is not fully understood, but it is notably enriched in presynaptic regions where it interacts with vesicles and membranes [2]. In humans, the SNCA gene encodes *α*Syn, a 140-amino-acid intrinsically disordered protein, a protein that lacks a stable three-dimensional structure in its native form and can adopt various shapes depending on its environment and interactions with other molecules [3,4].

*α*Syn gained significant attention due to its association with neurodegenerative disorders, particularly Parkinson’s disease (PD) [2]. Beyond PD, *α*Syn pathology is also implicated in other neurodegenerative disorders collectively referred to as synucleinopathies, which include dementia with Lewy bodies (DLB) and multiple system atrophy (MSA). In PD, *α*Syn undergoes a process of misfolding and aggregation, forming insoluble protein aggregates (Lewy bodies), a hallmark pathological feature of the disease. It is believed that these aggregated forms of *α*Syn disrupt normal cellular processes and contribute to the degeneration of dopamine-producing neurons in the brain, leading to the motor and non-motor symptoms characteristic of PD. While the majority of PD cases are sporadic (approximately 85%), there are rare familial forms associated with the *α*Syn-encoding SNCA gene [5]. One group of familial forms involves mutations in SNCA. The first discovered mutation, found in several Italian and Greek families, leads to the formation of the *α*Syn A53T (*α*Syn-A53T) variant, known for its increased aggregation propensity compared to normal *α*Syn [6,7]. The second group of familial PD forms connects SNCA to PD through an alternative mechanism, namely triplications of the SNCA locus that lead to increased accumulation and aggregation of *α*Syn [8].

Both (over)expression and mutations of an intrinsically disordered protein such as *α*Syn can lead to alterations in protein aggregation pattern, which challenges cellular protein homeostasis (proteostasis) and often causes proteotoxic stress [9,10]. All eukaryotes possess three integrated but distinct subsystems for combating proteotoxic stress: chaperones and two protein degradation systems (the ubiquitin/proteasome system and autophagy) [10,11]. The first line of defense against the aggregation of improperly folded and unfolded proteins is the chaperones, most of which belong to the heat shock protein (Hsp) family and are subclassified based on the mass of their monomeric subunits (e.g., Hsp70 family and the Hsp20 family) [11]. If the chaperone system is overwhelmed by the amount of damaged or misfolded proteins, these aberrant proteins are directed to the ubiquitin/proteasome system (UPS) or autophagy for degradation [12,13]. Both UPS-dependent and autophagy-dependent degradation of *α*Syn have been described in animal models [14,15].

In addition to the proteostatic stress, the aggregation of *α*Syn is linked to the dysregulation of iron metabolism, which is believed to contribute to or enhance the disease syndrome [16,17]. Two hallmarks of iron dysregulation in PD are elevated iron content and reduced ferritin levels [16,18]. Ferritins are iron storage proteins present in all biological kingdoms. Although an amino acid sequence comparison of ferritin subunits revealed that plant and animal ferritins have evolved from a common ancestor gene, there are two major differences between plant and animal ferritins [19,20]. The first difference concerns their subcellular localization: whereas ferritins are mostly cytosolic soluble proteins in animal cells, they are mainly found in the stroma of various plastids in plants [19,20]. The second difference between plant and animal ferritins concerns the regulation of their synthesis in response to excess iron: whereas this regulation occurs mainly at the translational level in animals, the regulation is transcriptional in plants [21]. Additionally, the expression of plant ferritin is not modulated only by iron surplus, but it is also regulated by oxidative stress, drought, cold, light intensity, and pathogen attack [22]. This has led to the suggestion that plant ferritins primarily function to sequester iron, hindering the initiation of cascading redox reactions rather than storing iron for future metabolic use [22]. This hypothesis is supported by the finding that, in plants, the rapid increase in ferritin transcript levels induced by iron excess can be counteracted by antioxidants [23].

Due to its crucial involvement in some neurodegenerative diseases, *α*Syn has become a primary focus of research aimed at devising strategies to hinder its aggregation or alleviate its adverse cellular effects. A promising avenue involves the identification of small molecules with anti-*α*Syn aggregation or stress-alleviating properties. Currently, a number of small-molecule inhibitors of *α*Syn aggregation from synthetic sources have been discovered, mostly through high-throughput screening strategies (e.g., [24–26]). However, plants offer another screening palette for the identification of aggregation inhibitors, expanding the potential pool of compounds that may hold therapeutic promise. In plants, defense against cell-damaging agents is commonly accomplished through the synthesis of diverse small bioactive molecules, collectively known as secondary metabolites [27–29]. These secondary metabolites, often specific to particular taxonomic groups, play a crucial role in protecting plants from various biotic and abiotic stressors, rather than directly participating in plant growth, development, and reproduction [30].

Hence, while both plants and animals have proteostatic and iron homeostasis regulatory mechanisms, plants uniquely possess the ability to synthesize intricate molecules as a defense against cytotoxic stimuli, such as the aggregation of intrinsically disordered proteins. Given that, we hypothesized that if the expression of the human *α*Syn-A53T protein in plant cells triggers both proteostatic defense mechanisms and alters iron homeostasis, then it is likely to trigger the synthesis of species-specific secondary metabolites that aid in defending cells against *α*Syn-induced dysregulation of cellular homeostasis. Our findings in four medicinal plants with documented neuroprotective effects—*Lobelia cardinalis*, *Polygonum multiflorum*, *Artemisia annua*, and *Salvia miltiorrhiza* [31–36]—confirm that *α*Syn-A53T expression indeed leads to proteostatic stress and iron homeostasis dysregulation. Thus, this approach yields plant models with a molecular signature of synucleinopathies, which may enable the discovery of novel neuroprotective natural products and/or the development of plant-based bioassays for potentially identifying drugs for treating or altering the progression of synucleinopathies.

## 2. Materials and Methods

### 2.1. Plant Materials

*L. cardinalis*, *A. annua*, and *S. miltiorrhiza* seeds were surface sterilized with 4% PPM^™^ (Plant Cell Technology, Washington, DC, USA) for ~16 h with constant agitation (180 rpm), followed by three rinses with sterile water. Seeds were cold–moist stratified for 14 days with 1 μM gibberellin A3 (GA3; Sigma-Aldrich, St. Louis, MO, USA) and sown on plates with Murashige and Skoog medium (MS, pH 5.7; [37]; Phytotech, Lenexa, KS, USA) supplemented with 3% sucrose and solidified with Gelrite (0.3%). Seedlings grown in controlled environmental growth chambers at 24 °C under a 16 h light/8 h dark photoperiod at 80 μmol m^−2^ s^−1^ were used for transformation. *P. multiflorum* seeds, procured from diverse sources, yielded seedlings with inconsistent and widely varied leaf and stem morphologies, leading to a general lack of trust in their authenticity. Instead, we obtained seedlings from Stricly Medicinal Seeds (https://strictlymedicinalseeds.com/; accessed on 17 May 2024). The supplied plants were vines with a stem twining from right to left and an alternate arrangement of cuneate leaves, which are gross morphological characteristics of *P. multiflorum* plants [38]. Seedlings were transplanted into 6.5-inch pots and kept in a control environmental chamber under continuous light (22 °C and a light intensity of 80 μmol m^−2^ s^−1^). Prior to transformation, young and mature leaves were harvested, washed in running tap water for 30 min, and surface sterilized by immersion in 70% ethanol (5 min) followed by a 30 min long incubation in 30% commercial bleach.

### 2.2. Genetic Construct

To generate human *α*Syn-A53T expression hairy root lines, a full-length hSNCA cDNA clone (based on NM_000345) was synthesized by GeneArt^™^ Services (Thermo Fisher Scientific, Cincinnati, OH, USA) and recombined by BP reaction into the pDONR221 vector. The cDNA was then transferred from the resulting pENTR-SNCA mutant clone into the pEarleyGate100 binary vector by LR reaction using the Gateway protocols (Thermo Fisher Scientific). pEarleyGate100 carries a Basta (DL-Phosphinothricin or glufosinate-ammonium; Phytotech) resistance gene for selection of transformants [39]. The resulting construct and the empty pEarleyGate100 binary vector were introduced into *Agrobacterium rhizogenes* strain R1000 using the heat-shock protocol optimized to reduce the generation of antibiotic-resistant *A. rhizogenes* clones [40].

### 2.3. Generation of Transgenic Hairy Root (HR) Lines

*L. cardinalis*, *A. annua*, and *S. miltiorrhiza* were transformed following the same protocol. In brief, leaves and cotyledons of two-month-old seedlings were excised, cut into 0.25 cm^2^ pieces, and used as explants for the transformation. *A. rhizogenes* strains containing the empty binary vector or pEarelyGate100-hSNCA A53T construct were grown for 48 h in LB medium supplemented with kanamycin at 28 °C and 180 rpm. The freshly cut explants were submerged into a bacterial suspension that contained 100 μM acetosyringone (Sigma) and incubated for 20 min. The excess bacteria suspension was removed by blotting onto sterile Whatman filter paper. The explants were then transferred to the MS media and incubated in darkness for 3 days. After solid-media co-culture with *A. rhizogenes*, explants were rinsed with sterile water, blotted dry and transferred onto hormone-free transformation media (MS solidified with 0.3% Gelrite supplemented with 3% sucrose, 500 mg/L cefotaxime (TCI) and 40 mg/L Basta). The explants were kept in controlled environmental growth chambers at 24 °C in the dark. HRs were excised from the explants, transferred to MS plates with cefotaxime and Basta, and sub-cultured every three weeks onto plates with a halved dose of the antibiotic.

Although previous studies reported protocols for the transformation of *P. multiflorum* (e.g., [41]), we could not obtain transgenic roots even after extensive adjustments of most of the variables. Instead, we used sonication-assisted Agrobacterium transformation (SAAT), an approach used when there is a need to increase the efficiency of transformation in low-susceptibility or non-susceptible plant species [42–44]. SAAT transformation was performed using leaf discs of sterilized, in vitro grown plants following a published protocol [45]. In brief, the sterilized tissue was sonicated for 5, 10, or 20 s in 15 mL of D40 medium (MS salts, B5 vitamins, 40 mg/L 2,4-D, 6% sucrose; pH 7.0), in which *A. rhizogenes* strains were diluted to the OD600 of 0.2. The leaf tissue (unwashed) was transferred to D40 media without antibiotics for a two-day-long solid-media co-cultivation in the dark. Explants were then transferred to D40 media 500 mg/L cefotaxime and kept in the growth chamber. Three weeks after infection, leaf discs sonicated for 20 s and maintained in low light on MS media with cefotaxime started developing HRs. HRs were excised from the explants, transferred to MS plates with cefotaxime, and sub-cultured every three weeks onto plates with a halved dose of the antibiotic.

### 2.4. Anthocyanin Measurements

For anthocyanin extraction, root tips of approximately 5 mm in length were removed, weighed, placed in acidic methanol, and kept at 4 °C in the dark with regular agitation, following an established procedure [46]. Anthocyanin levels were quantified using a Nanodrop 2000 (Thermo Fisher Scientific). Each HR sample was measured in six replicates, and a minimum of five roots per line were tested.

### 2.5. Treatments

To test the relative importance of the UPS and autophagy in the degradation of *α*Syn-A53T in the HR OEX lines, apical parts of HRs were excised and treated with either DMSO (control), MG132 (100 μM; [47]; Enzo, Farigdale, NY, USA), or the autophagy inhibitor wortmannin (10 μM; [48]; Enzo). After a 24 h long incubation, DMSO-treated, MG132-treated, and wortmannin-treated HRs were blotted dry, snap-frozen, and used for protein extraction and immunoblotting analyses as described [49]. To assess the effects of short-term iron treatments, we treated HR cultures with a 20 mM aqueous solution of iron(II) ammonium sulfate for 2 h at room temperature. Samples were then blotted dry, snap-frozen, and used for protein extraction.

### 2.6. Immunoblotting Analyses

For protein extraction, HR segments were weighed, and approximately 100 mg per sample was transferred to tubes with zirconium beads and frozen in liquid nitrogen. Five volumes of 2X Laemmli sample buffer were added to each sample, and tissues were disrupted in a BeadBug bead beater (MidSci, Feton, MO, USA) for a total of 4 min at 3000 rpm. Samples were spun for 10 s, heat denatured at 95 °C, and centrifuged at room temperature for 15 min at maximum speed. After the debris was pelleted, proteins were separated on 4–20% SDS-PAGE gels (Bio-Rad, Hercules, CA, USA) and transferred to nitrocellulose membranes. Membranes were blocked using 10% fat-free milk and incubated with primary antibodies diluted in PBS containing 0.2% Tween-20 (PBST). Primary antibodies used were anti-*α*Syn antibody raised against the full-length recombinant protein with the epitope mapped to amino acids 118–123 (1:10,000 dilution; abcam ab138501; Abcam, Bosto, MA, USA), anti-Hsp17.6 (1:5000 dilution; Agrisera Product No. AS07 254; Agrisera, Reveljgränd 4, 903 47 Umeå, Sweden), anti-Hsp17.6 (1:1000; Agrisera AS07 254), anti-Hsp70 (1:5000; Agrisera AS08 371), anti-glutamine synthase (GS) (1:1000, Agrisera AS01 018), anti-alphatubulin (TUA) (1;10,000; Millipore-Sigma T5168: Burligton, MA, USA), anti-NBR1 (1:1000, Agrisera AS14 2805), anti-polyubiquitin (Ub) (1:1000, Santa Cruz Biotechnology sc-58448; Santa Cruz, CA, USA), anti-ferritin (1:1000, Agrisera AS15 2898), and anti-FIT (1:1000, PhytoAB, PHY0939A; San Jose, CA, USA). Secondary antibodies used were goat anti-mouse IgG-HRP (Santa Cruz Biotechnology, 1:1000 dilution) and goat anti-rabbit IgG-HRP (Santa Cruz Biotechnology, 1:1000). Immunoblots were developed using SuperSignal West Femto substrate (Thermo-Pierce, Rockford, IL, USA) using a ChemiDoc XRS molecular imager and quantified using Quantity One^®^ software Version 4.5 (Bio-Rad) or ImageJ Version 1.53r (https://imagej.net/ij/).

### 2.7. Ferric Chelate Reductase (FCR) Activity Measurements

HR cultures passaged onto fresh media and cultivated in light for three weeks were used as a source of root apical segments. Tissue was rinsed with distilled water, blotted dry, weighed, and used for the ferric chelate reduction assay following a described protocol [50]. In brief, root segments were immersed into 1 mL of the assay solution (0.1 mM Fe-EDTA, 0.3 mM FerroZine; both from Sigma-Aldrich, St. Louis, MO, USA) and incubated in the dark for 25 min at 24 °C. The absorbance of the assay solution was then measured at 526 nm and normalized to root weight.

### 2.8. Experimental Procedure Overview and Statistical Analyses

**Figure 1.**
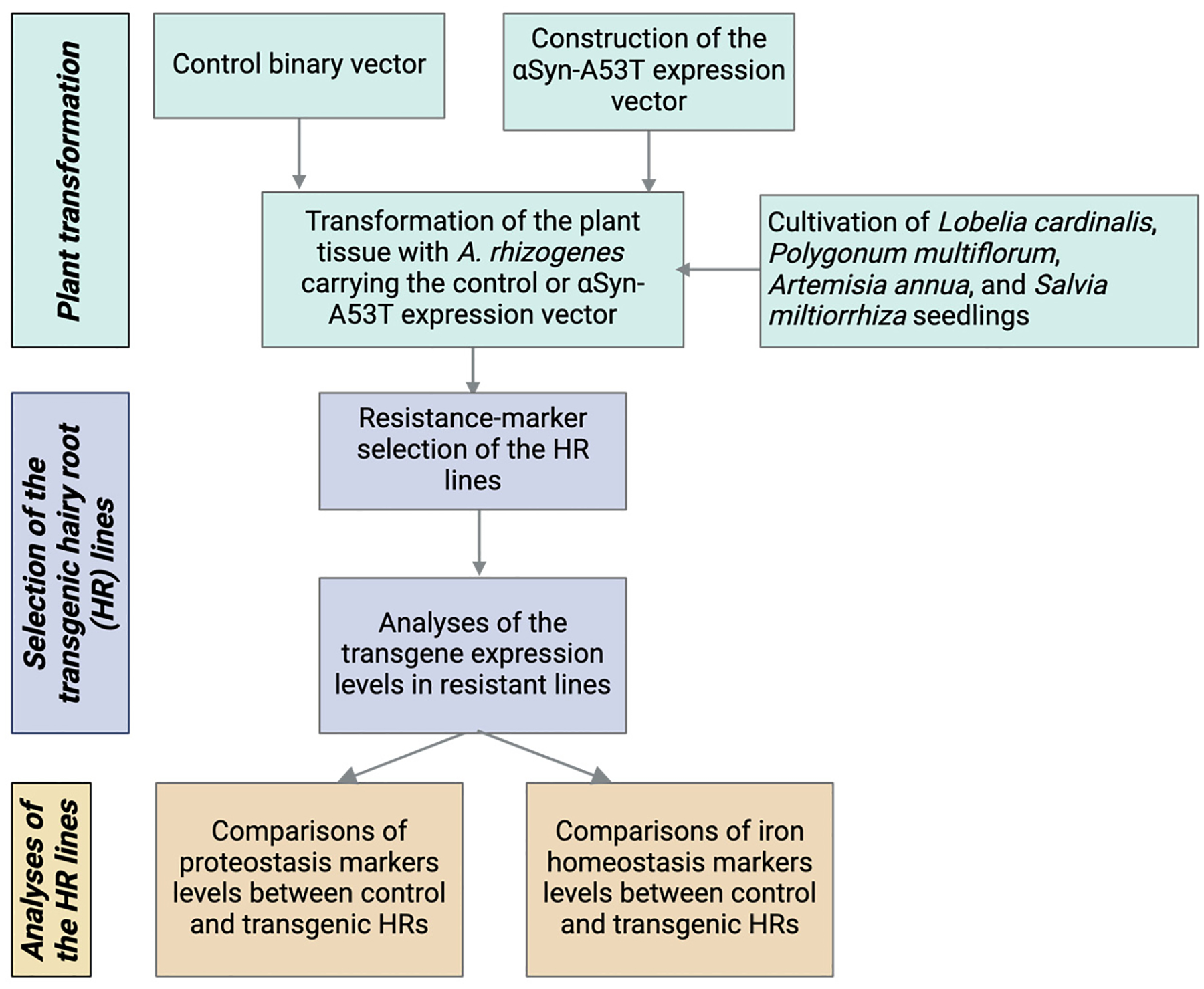
Overview of the experimental procedure (refer to the Section 2 for details).

An overview of our approach is illustrated in Figure 1. Descriptive statistics, plotting, and hypothesis testing were performed using Prism 9.3 (GraphPad Software Inc., La Jolla, CA, USA). All data are presented as mean ± SD. The statistical tests used to analyze the data, the size of tested sample sets, and the number of biological replicates are stated in the Section 3 or figure legends.

## 3. Results and Discussion

### 3.1. Growth Patterns of the Hairy Root (HR) Cultures

HR cultures were generated with varying levels of difficulty and exhibited distinct morphologies among the different plant species. *L. cardinalis*, *A. annua*, and *S. miltiorrhiza* were easily transformed using the basic HR generation protocols. *P. multiflorum* proved to be more recalcitrant, and HR cultures were obtained only after the SAAT protocol was used (see Section 2).

**Figure 2.**
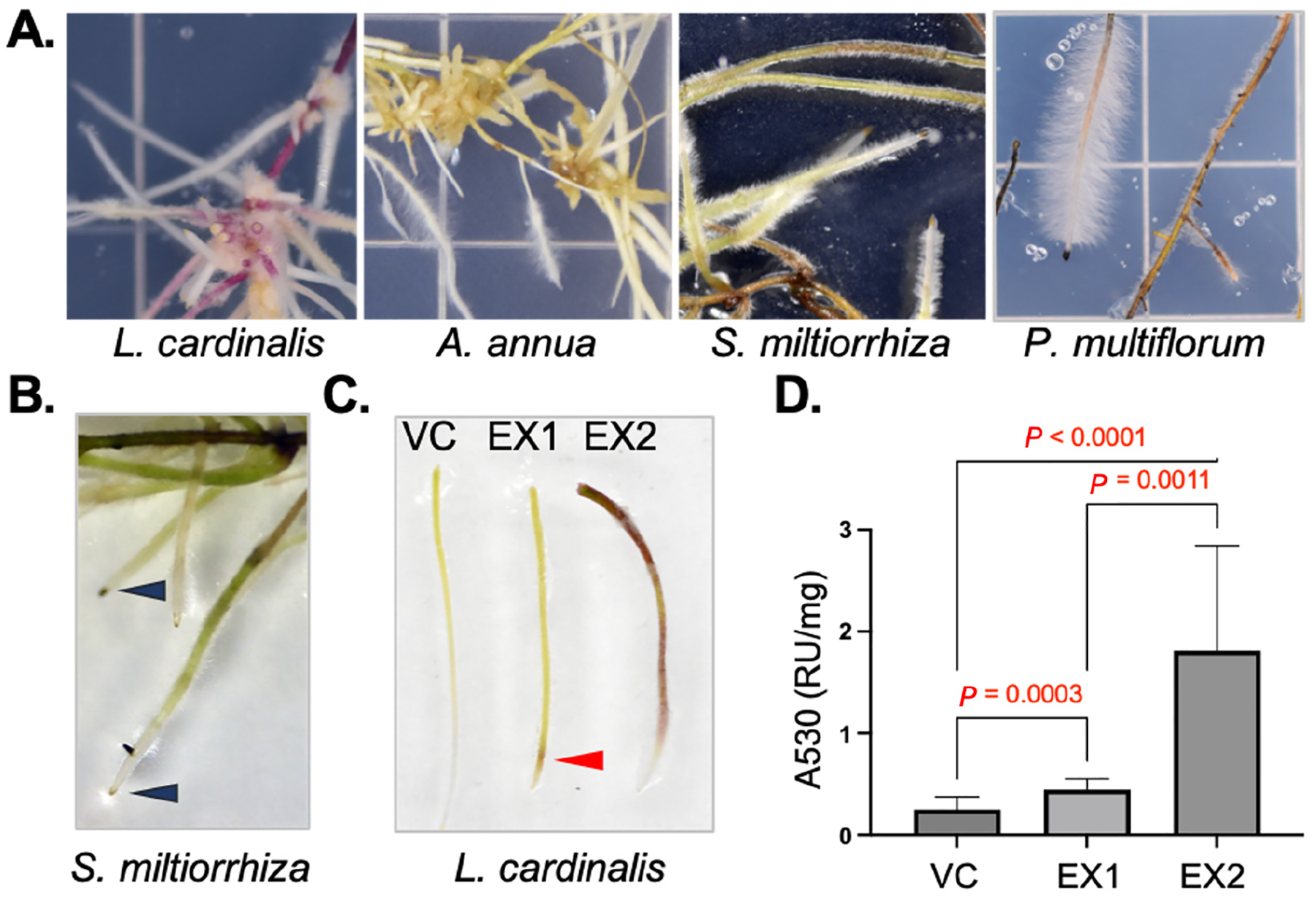
Basic growth patterns of generated HR cultures. (**A**) Representative photographs of root tips of cultures passaged onto MS media and grown for three months. (**B**) Image of *S. miltiorrhiza* root tip region emphasizing the accumulation of dark pigments in the root cap (arrowheads). (**C**) Representative root tip regions of the *L. cardinalis* vector control (VC) and two *α*Syn-A53T expression (EX) lines, illustrating the difference in the accumulation patterns of anthocyanins. Arrowhead points to the region accumulating the anthocyanins in EX1 line. (**D**) Anthocyanin accumulation in *L. cardinalis* VC and two *α*Syn-A53T EX lines is presented as absorbance at 530 nm (A530) per milligram fresh weight. Data are expressed as mean ± SD (n ≥ 20; significance of the difference calculated with one-way ANOVA with Tukey’s post-test is indicated).

HR lines derived from *L. cardinalis*, *A. annua*, *S. miltiorrhiza*, and *P. multiflorum* had unique morphologies (Figure 2A), and with the exception of *L. cardinalis* HR lines (see Section 3.2), vector control and *α*Syn-A53T expression lines of each species did not visibly differ. *L. cardinalis* HR cultures were most prolific, were green or purple, and had less pronounced root hairs compared to HR cultures of the other species. The root tips of *A. annua* HRs were white with pronounced root hairs, whereas the older parts accumulated polyphenols (i.e., became darker) and thickened with prolonged cultivation. *S. miltiorrhiza* was morphologically similar to *A. annua* with the exception that fewer polyphenols accumulated in older cultures and fewer polyphenols were released into the growth media with prolonged cultivation (Figure 2B). *P. multiflorum*-derived HRs had a distinct pale root growth zone with a profusion of long root hairs. Older parts of the *P. multiflorum*-derived HRs did not thicken but rather accumulated dark pigments and necrotized. Extended cultivation on MS media proved detrimental, leading to the transformation of these HRs into calli. Of all the HR lines, the *P. multiflorum* lines were particularly difficult to work with. The scarcity of root branching, the stunted lateral root growth, and the fragile, brown, and brittle nature of mature roots, which were susceptible to calli formation, constrained the quantity of tissues accessible for analysis (Figure S1 in Supplementary Materials).

### 3.2. Expression of αSyn-A53T in L. cardinalis HRs Leads to Proteostasis Dysregulation

**Figure 3.**
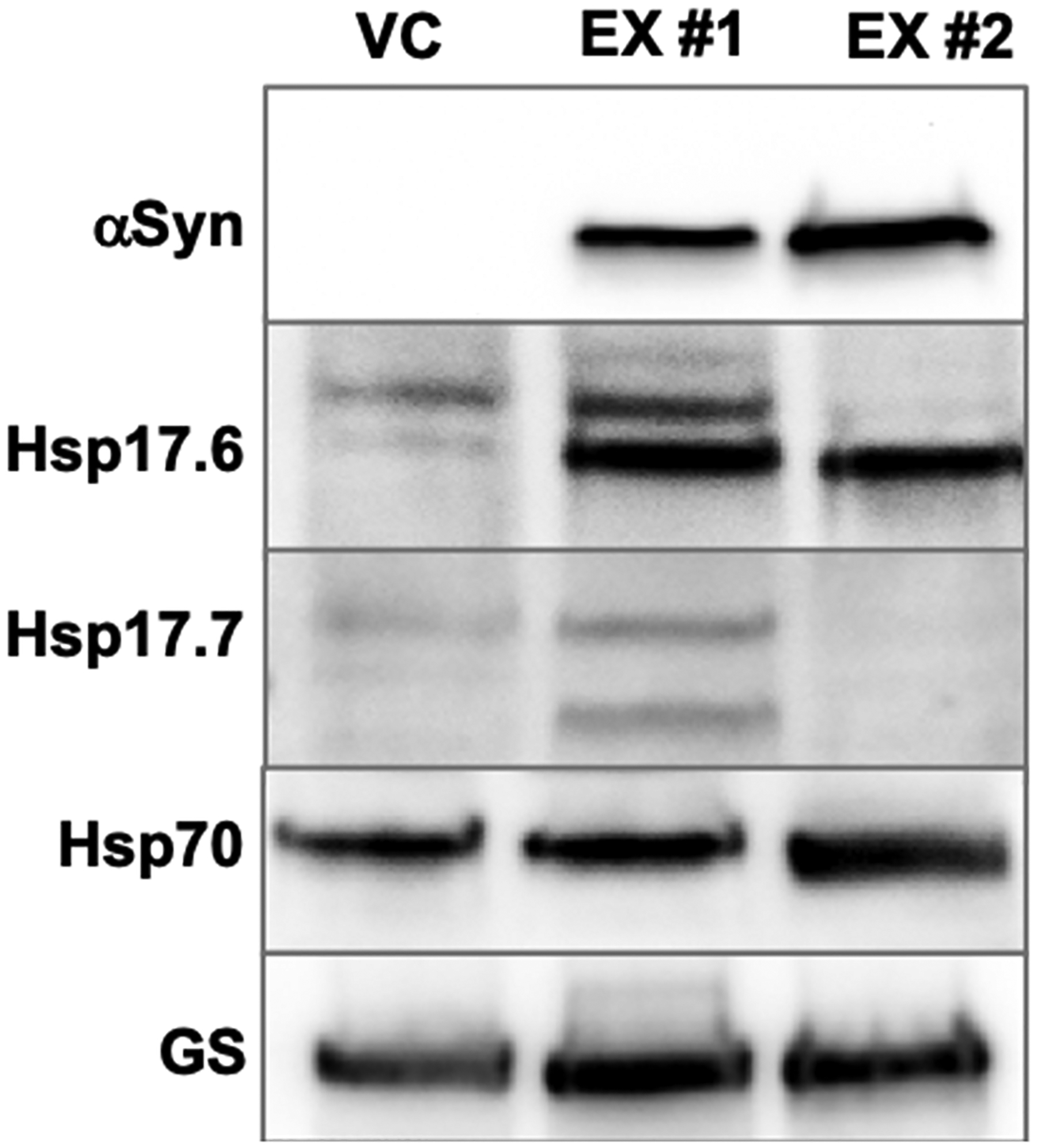
Expression of *α*Syn-A53T in *L. cardinalis* HRs leads to proteostatic stress. Representative immunoblots illustrating the levels of *α*Syn-A53T monomer, small heat shock proteins (Hsp17.6 and Hsp17.7), and Hsp70 in the vector control (VC) and expression lines (EX) #1 and #2. Apical regions of the HRs grown on fresh media for three weeks were excised, weighed, and used for protein isolation and immunoblotting. Glutamine synthase (GS) is shown as a loading control.

**Figure 4.**
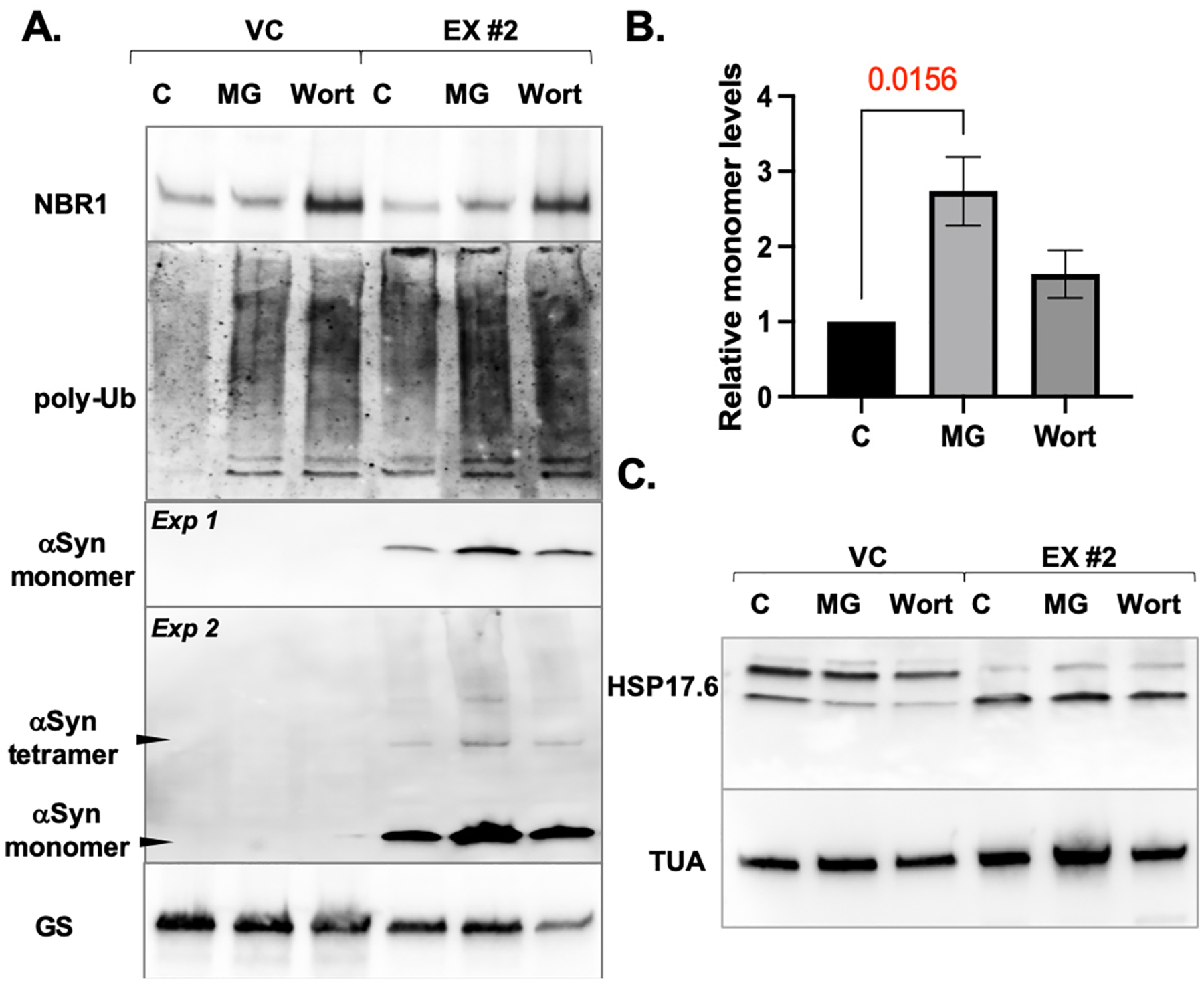
Effects of proteasome and autophagy inhibitors on the accumulation of the proteostatic components in *L. cardinalis* HRs. (**A**) Representative immunoblots illustrating the levels of the autophagy receptor NBR1, poly-ubiquitinated proteins (poly-Ub), and *α*Syn-A53T in the vector control (VC) and the *α*Syn-A53T EX #2 line. Apical regions of the HRs grown on the fresh media for three weeks were excised, incubated in DMSO control (C), MG132 (MG, 100 μM), or wortmannin (Wort, 10 μM) for 24 h, and then used for immunoblotting. Membranes were probed with anti-glutamine synthase (GS) antibodies as a loading control. *Exp1* and *Exp2* refer to a shorter and longer exposure of the immunoblot. (**B**) Quantification of signal intensity of *α*Syn-A53T monomers. The relative intensity levels are presented as mean and SD (n = 3), with the mean strength of the control signal assigned the value of 1. (**C**) Representative immunoblot illustrating the levels of the Hsp17.6 protein in VC and the EX #2 lines treated with DMSO (C), MG132 (MG, 100 μM), or wortmannin (Wort, 10 μM). Membrane probed with anti-*α*-tubulin (TUA) antibodies is shown as loading control.

*L. cardinalis* HR cultures, being the most prolific and least susceptible to tissue necrosis, were selected for comprehensive analyses of the effects of *α*Syn-A53T expression. Among *L. cardinalis* transgenic HR lines, we noted variations in color and selected two of these variable lines, *α*Syn-A53T expressors #1 and #2 (EX #1 and #2), for further analyses (Figure 2C). Given that anthocyanins are the most prevalent purple pigment in plants [51], we measured the total anthocyanin content in these two lines and confirmed that the purple-colored HR line was indeed an anthocyanin hyperaccumulator (Figure 2D). Immunoblotting analyses utilizing anti-*α*Syn antibodies validated the expression of *α*Syn-A53T in both lines and demonstrated that the detectable form of *α*Syn-A53T was predominantly the monomeric variant (Figures 3 and 4A). Notably, the HR line with high anthocyanin levels expressed *α*Syn-A53T monomer at a higher level (~2-fold) compared to the line with lower anthocyanin content.

The overexpression of *α*Syn and the disruption of proteostasis are closely connected processes in neurodegenerative diseases, such as PD [52]. Since proteostasis mechanisms are operational in all eukaryotes [9,10], we posited that expressing *α*Syn-A53T in plants would also induce proteostatic stress. To assess this, we compared the levels of various components of the proteostasis maintenance systems in the control and *α*Syn-A53T-expressing HR lines (Figure 3). Beginning with the chaperones, we tested the levels of Hsp17.6 and Hsp17.7, two members of the small Hsps belonging to the Hsp20 subfamily [53,54]. In *L. cardinalis* HR cultures, the Hsp17.6 antiserum detected multiple proteins and revealed that the abundance of the lowest molecular weight isoform was markedly increased in *α*Syn-A53T-expressing HR lines when compared to the vector control (3.4 ± 0.3- and 2.5 ± 0.2-fold increase in EX #1 and EX #2 lines). Interestingly, the abundance of different Hsp17.6 isoforms differed between the higher (EX #2) and lower (EX #1) *α*Syn-A53T-expressing HR lines. As small Hsps interact with partially folded proteins to stabilize them and prevent aggregation [53,54], the increase in total Hsp17.6 levels within *α*Syn-A53T HR lines implies heightened proteostatic stress. Additionally, the line-specific variations in Hsp17.6 levels appear to reflect the stress intensity, with the highest *α*Syn-A53T-expressing line likely invoking defensive mechanisms beyond small Hsps. Interestingly, small Hsps were shown to exhibit a notable impact on *α*Syn aggregation, positioning them as promising candidates for synucleinopathy therapeutics [55]. For example, human Hsp27 has shown promise in alleviating *α*Syn-induced neurotoxicity in cell cultures, displaying higher effectiveness in combating *α*Syn aggregation compared to other Hsps (e.g., Hsp70) [55]. Our findings indicate that the involvement of small Hsps in combating *α*Syn overaccumulation is evolutionarily conserved.

We next compared the abundance of Hsp17.7 between control and EX lines. The anti-Hsp17.7 antibody recognized two proteins in *L. cardinalis* HR, with the higher-molecular-weight species aligning with the predicted molecular weight (Figure 3). Notably, Hsp17.7 levels were increased in the low-level *α*Syn-A53T-expressing line and significantly reduced in the high-level *α*Syn-A53T-expressing HR line. This expression profile reinforces our conclusion that while a lower level of *α*Syn-A53T expression already triggers proteostatic stress, the increase in transgenic protein load necessitates the recruitment of proteostatic mechanisms that act beyond the stabilization of partially folded proteins.

Next, we tested the levels of Hsp70 (Figure 3). In contrast to Hsp17.6 and Hsp17.7, which functionally belong to the holdase-type chaperones that interact with partially folded proteins in an ATP-independent manner, Hsp70s are foldase-type chaperones that facilitate the folding of protein intermediates to their native folded state in an ATP-dependent manner [56,57]. The rise in the Hsp70 level in the higher *α*Syn-A53T expression line, in contrast to the control and low-level *α*Syn-A53T HR line, suggests that energy-dependent chaperone subsystems needed to be activated in this line.

In summary, the immunoblotting results confirmed that the expression of *α*Syn-A53T in HR cultures triggers a dose-dependent induction of proteostatic stress.

### 3.3. αSyn-A53T Expressed in L. cardinalis HRs Is a Degradation Target of the UPS

In animal models, both UPS-dependent and autophagy-dependent degradation pathways for *α*Syn have been documented [14,15]. For example, while some studies employing a pharmacological proteasome inhibition approach demonstrated *α*Syn degradation by the proteasome, others reported no significant accumulation of *α*Syn or showed that intermediate oligomeric forms of *α*Syn were degraded by the UPS whereas monomer and higher-order *α*Syn were not [58–62]. The current belief is, therefore, that the UPS may be responsible for the degradation of a fraction of *α*Syn, and the identity and the size of the *α*Syn fraction targeted to the proteasome may depend on the experimental system and conditions [14]. Similarly, both upregulation of autophagic flux and inhibition of autophagy were described in *α*Syn-overexpressing cell lines and animal models [15]. Collectively, these studies show that high levels of wild-type, mutant, and modified *α*Syn species interfere with both proteasome-dependent degradation and autophagy, and how these two basic proteostatic systems are affected depends on several variables (e.g., cell culture type, type of model organism, experimental conditions) [15].

To test the relative importance of the UPS and autophagy in the degradation of *α*Syn-A53T in the HR lines, we treated the *L. cardinalis* control line and the higher-level *α*Syn-A53T-expressing line with 100 μM of the proteasome inhibitor MG132 or 10 μM of the autophagy inhibitor wortmannin for 24 h (Figure 4). To validate the efficacy of the treatments, we assessed the levels of NBR1 and polyubiquitinated proteins (Figure 4A). NBR1 functions as a selective autophagy receptor, directing the NBR1/cargo protein complex to the autophagy pathway for degradation [63,64]. Inhibition of autophagy is expected to increase NBR1 levels, which we observed in both control and expression lines. Next, we examined the levels of polyubiquitinated protein (i.e., proteins covalently modified with chains of the small protein ubiquitin, which serves as a sorting tag for UPS degradation as well as for directing some of the aggregated protein cargo for autophagic processing [12,64]). Inhibiting both the proteasome and autophagy should lead to elevated polyubiquitinated protein levels, a treatment response observed in both control and *α*Syn-A53T lines. However, in the *α*Syn-A53T line, polyubiquitinated proteins significantly accumulated without treatment, and the increases induced by MG132 and wortmannin were insignificant compared to those in the control line (Figure 4A). This suggests that *α*Syn-A53T expression increases polyubiquitination rates or decreases the degradation rates of polyubiquitinated proteins, possibly due to overload in the UPS and autophagic pathways, both indicative of heightened proteostatic stress. The *α*Syn-A53T-induced buildup of polyubiquitinated proteins in plant cells echoes the finding that in PD, Lewy bodies contain high levels of ubiquitin, similarly indicating increased ubiquitination rates, a decline in the degradation of ubiquitinated cargo, or both [65].

Analysis of *α*Syn-A53T levels revealed an increase in monomeric and oligomeric forms only in response to treatment with the proteasome inhibitor, suggesting the UPS as the main degradation pathway (Figure 4A,B). There were no significant differences in Hsp17.6 levels between vector control and *α*Syn-A53T expression HR lines in response to MG132 or wortmannin (Figure 4C).

### 3.4. Expression of α-Syn-A53T Results in Disruption of Iron Homeostasis

The levels of the iron-binding protein ferritin are considered a reliable indicator of iron status in both animals and plants, with elevated ferritin levels associated with increased iron content [17,21,66]. Ferritin-dependent sequestration of iron is instrumental in limiting the levels of free reactive iron in cells, thereby preventing iron-mediated oxidative stress [19,67]. However, ferritin regulation in PD is atypical. In PD, *α*Syn aggregation is linked to an increase in iron content in synaptic neurons, surprisingly accompanied by ferritin downregulation. This unusual alteration of iron and ferritin levels is believed to be a significant contributor to the disease, as the resulting rise in oxidative stress further amplifies *α*Syn aggregation [16,68].

**Figure 5.**
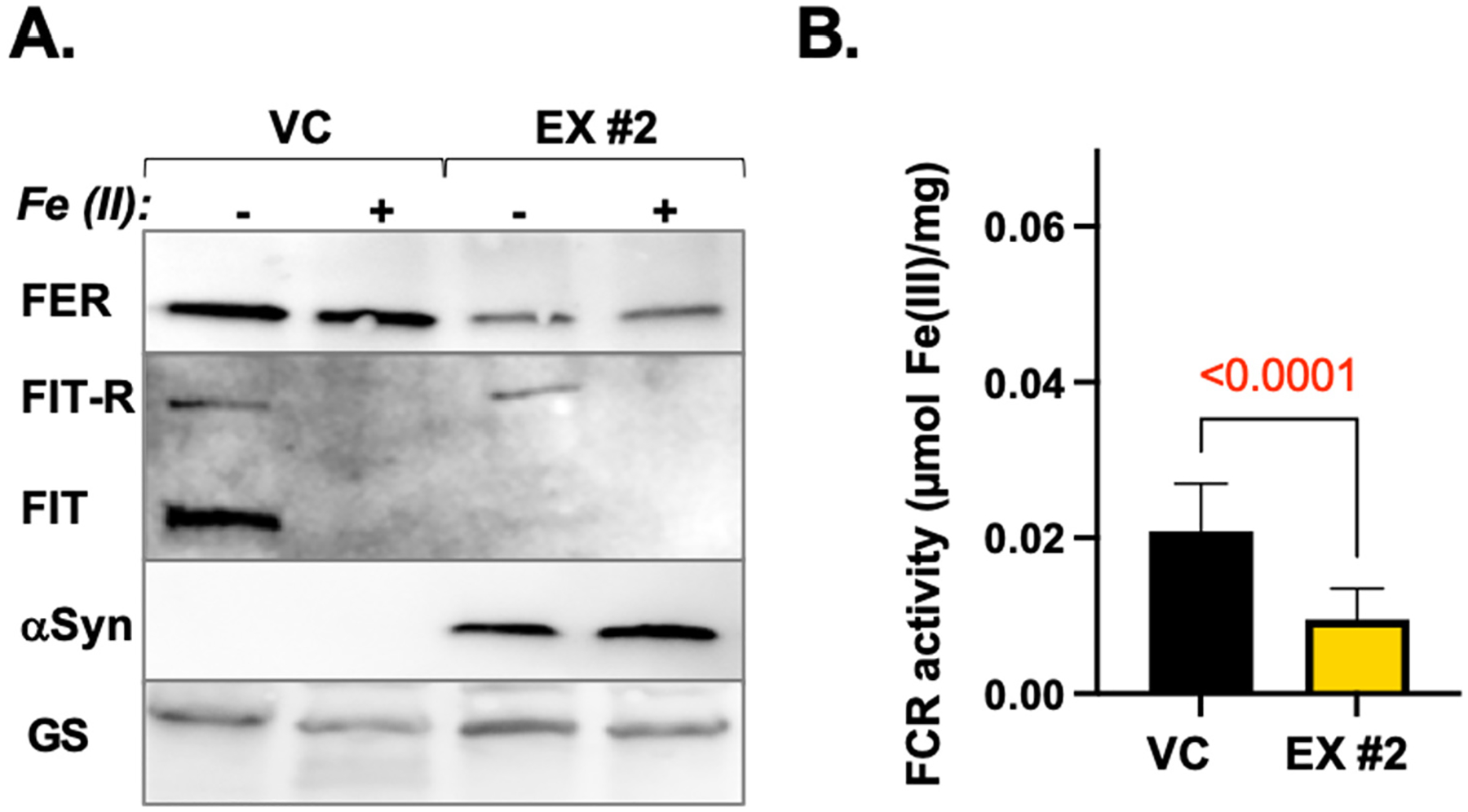
Dysregulation of ferritin and FIT levels and reduction in FCR activity in *L. cardinalis* HRs expressing *α*Syn-A53T. (**A**) Representative immunoblots display ferritin (FER), FIT, and *α*Syn-A53T levels in vector control (VC) and *α*Syn-A53T-expressing line #2 (EX #2). HR cultures were treated with iron(II) ammonium sulfate for 2 h. The levels of glutamine synthase (GS) are shown as the loading control. A higher-molecular-weight band recognized by Arabidopsis anti-FIT antibodies is labeled as FIT-R (FIT-related). (**B**) Ferric chelate reductase (FCR) activity in the apical portions of the HRs of VC and EX #2 lines. Three separate roots from three different cultures per lane were harvested, pooled, weighed to normalize the data per fresh weight (in mg), and used for FCR measurements. The data are presented as mean ± SD (n = 3). The significance of the difference between the control and the EX line was calculated using a two-tailed unpaired *t*-test.

To investigate the effects of *α*Syn-A53T expression on iron homeostasis in HRs, we analyzed the levels of ferritin and the transcription factor FIT. FIT is a key regulator of the iron uptake system in plant roots as it orchestrates the upregulation of essential iron uptake genes [22,69,70]. During iron starvation, the expression of FIT increases, and as FIT protein accumulates, it is actively and continuously degraded by the UPS to prevent iron overload and its adverse effects [22,69–71]. To determine ferritin and FIT levels in HR cultures, apical root segments were treated with 20 mM aqueous solution of iron(II) ammonium sulfate for 2 h and used for immunoblotting analyses with anti-ferritin (FER), anti-FIT, and anti-*α*Syn antibodies (Figure 5A).

Ferritin levels were notably lower in the untreated *α*Syn-A53T-expressing line compared to the untreated vector control (0.4 ± 0.2 of the control levels; n = 3). Similarly, FIT levels in untreated HR cultures differed significantly, with the *α*-Syn A53T-expressing line showing a lower FIT level compared to the vector control. Given that FIT levels typically increase in response to iron deficiency, this suggests that *α*Syn-A53T expression in HR leads to iron accumulation, consistent with findings in human and animal systems [16]. In the treated cultures, ferritin levels did not increase in either the control or the *α*Syn-A53T expression line, aligning with previous studies indicating that short-term iron treatments do not significantly impact ferritin expression [72,73]. In contrast, this short-term iron treatment downregulated the iron-response regulator FIT in both lines, indicating that the HRs indeed detected a change in intracellular iron levels.

To explore another facet of iron metabolism in HR lines, we measured the activity of ferric chelate reductases (FCRs). Despite iron being the second most abundant metal on Earth, its primary form in the environment is the water-insoluble and metabolically inactive Fe^3+^ [74]. One strategy—known as Strategy I—that evolved in plants to allow the absorption of bioactive and water-soluble ferrous ions depends on ferric chelate reductases (FCRs). FCRs catalyze the transfer of electrons from cytosolic NADPH to extracellular ferric ions (Fe^3+^), generating ferrous ions (Fe^2+^) which are then transported across the plasma membrane by specific iron transporters [69,75]. Although no published studies currently focus on iron uptake and homeostasis in any of the plant species used to generate HR cultures, we hypothesized that, since all tested species are non-gramineous (and thus do not acquire iron from soils using the siderophore-based Strategy II [75]), FCR is an essential component of their iron uptake mechanisms.

Total FCR activity was significantly reduced in the *α*Syn-A53T-expressing line (Figure 5B). In Arabidopsis, an FCR isoform named FRO2 is considered the major Fe^3+^ chelate reductase, and its gene is transcriptionally induced by FIT1 [69,76]. Based on these data and assuming the existence of homologs of FRO2 and FIT1 in *L. cardinalis*, the decrease in FCR activity in the *α*Syn-A53T line aligns with the observed lower levels of FIT compared to the vector control.

These findings not only reinforce the conclusion that HRs expressing *α*Syn-A53T are valid plant models for synucleinopathy but also suggest that the molecular pathways underlying the associated iron homeostasis dysregulation might be universal across different biological kingdoms.

### 3.5. Dysregulation of Proteostasis and Iron Metabolism Is Observed in _α_Syn-A53T Expression Lines of Other Plant Species

**Figure 6.**
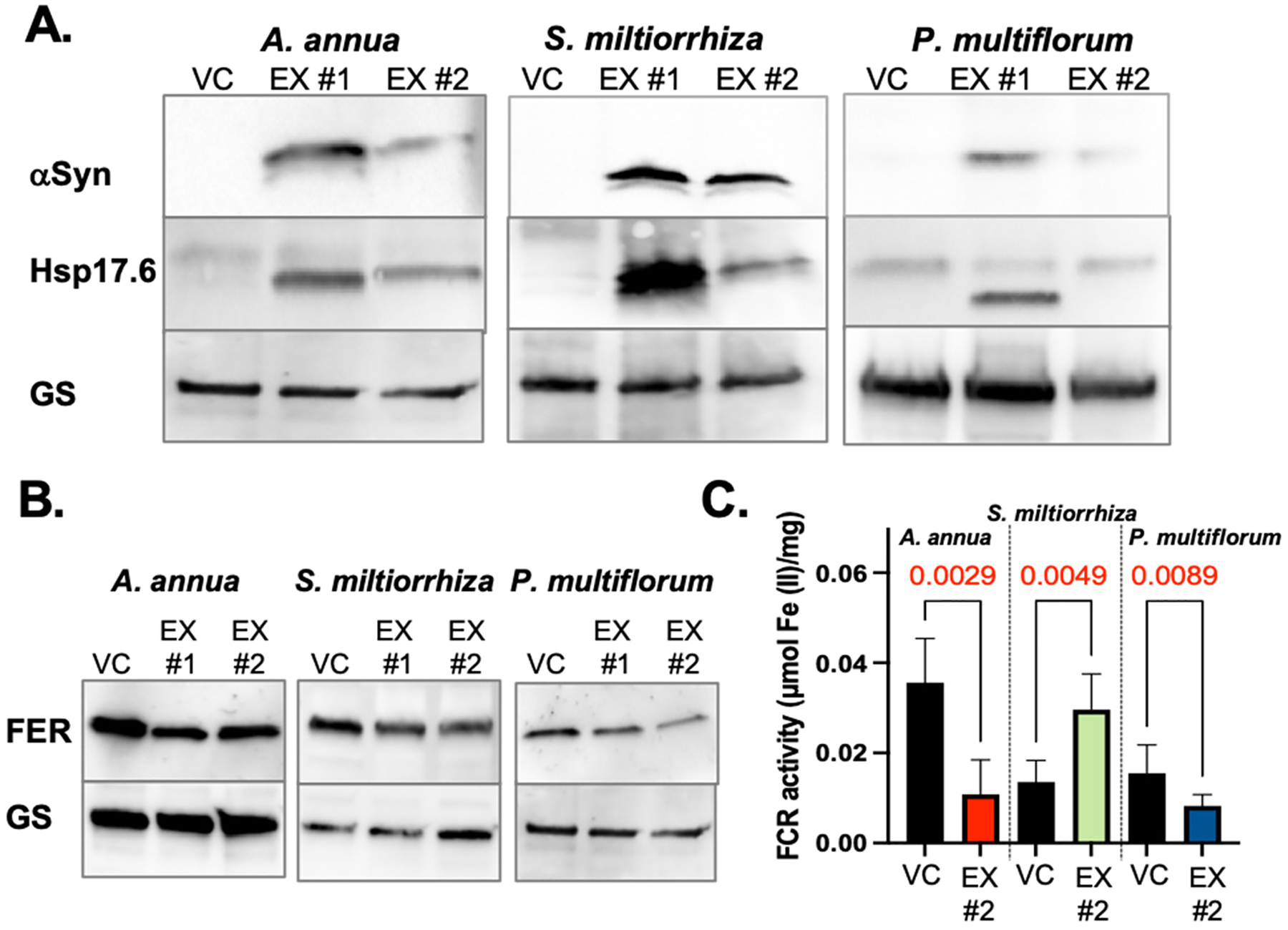
Effect of *α*Syn-A53T expression on proteostasis and iron homeostasis markers in *A. annua*, *S. miltiorrhiza*, and *P. multiflorum* HR cultures. (**A**) Representative immunoblots illustrating the levels of the *α*Syn-A53T monomer and Hsp17.6 in two independent *α*Syn-A53T expression (EX #1 and #2) HR cultures of the denoted species. Glutamine synthase (GS) levels are shown as a loading control. (**B**) Ferritin (FER) levels in HR cultures of different plant species. Glutamine synthase (GS) levels are shown as a loading control. (**C**) Ferric chelate reductase (FCR) activity in the apical portions of roots of the respective vector controls (VCs) and EX #2 lines of different plant species. Three separate roots of three different cultures per lane were harvested, pooled, and used for measurements. Data are presented as mean ± SD (n = 3; significance of the difference between the vector control of each species and the EX line was calculated using a two-tailed unpaired *t*-test).

Proteostatic imbalance and dysregulated iron homeostasis have been consistently observed across various systems related to *α*Syn overexpression or the expression of aggregation-prone *α*Syn mutant variants, including different animal models, actual Parkinson’s disease patient tissues, and *L. cardinalis* HRs. To assess whether these effects of *α*Syn can be extended to plants more broadly, we tested the abundance of Hsp17.6 and ferritin in HR lines of *Artemisia annua*, *Salvia miltiorrhiza*, and *Polygonum multiflorum*. To do so, we extracted total proteins from the respective vector control cultures as well as from two separate *α*Syn-A53T-expressing lines of each species and conducted immunoblotting analyses (Figure 6). Similar to *L. cardinalis*, changes in Hsp17.6 and FER levels were observed in all *α*Syn-A53T-expressing lines (Figure 6A,B). However, only changes in *A. annua* HRs closely resembled those observed in *L. cardinalis* (a dose-dependent increase in Hsp17.6 and a marked decrease in FER levels). The reduction in FER levels was less pronounced in both *S. miltiorrhiza* and *P. multiflorum*, and the increase in Hsp17.6 in *S. miltiorrhiza* was not correlated with the *α*Syn-A53T expression level. The FCR activity measured in the apical portions of roots of the vector control and *α*Syn-A53T expression lines of *A. annua* and *P. multiflorum* followed the same trend observed in *L. cardinalis*, with the strongest FCR downregulation observed in *A. annua* (Figure 6C). However, the FCR level in *S. miltiorrhiza* increased in response to *α*Syn-A53T expression.

We concluded that while the expression of *α*Syn-A53T in HRs of other tested plant species also resulted in an imbalance of protein and iron homeostasis, there are species-specific effects of *α*Syn-A53T expression. These effects may reflect the biochemical milieu of the plant cells of different species, determined by the stress defenses offered by secondary metabolites.

## 4. Concluding Remarks

A number of homeostatic changes have been observed in mice and other PD models expressing the human A53T mutant *α*Syn variant [77]. In mice, for example, the expression of A53T leads to alterations in dopamine metabolism and neurotransmission, dysregulation of iron homeostasis, impaired proteostasis and protein aggregation, disruption of mitochondrial function and dynamics, dysfunction in autophagy and lysosomal pathways, increased reactive oxygen species production, and dysregulation of calcium signaling and homeostasis [78–88]. While some of these homeostatic alterations are specific to animals, others involve pathways and mechanisms that are common to eukaryotes in general.

In this study, we discovered two homeostatic imbalances that are common to both animal and plant synucleinopathy models: proteostatic and iron homeostatic dysregulation. Although the plant model system presented here does not allow for the investigation of PD progression at the level of neurodegeneration, it offers opportunities to explore *α*Syn toxicity at the proteostasis and iron dysregulation levels, both of which are core components that trigger the onset of PD.

In animal cells, *α*Syn exists in an equilibrium between a soluble, intrinsically disordered state and a membrane-bound, partly *α*-helical state [89]. Soluble monomeric *α*Syn is a natively unfolded protein, assuming various conformational states [90]. Under normal conditions, soluble *α*Syn exists in a dynamic equilibrium between unfolded monomers and *α*-helically folded tetramers with a low aggregation propensity. Each conformational state of *α*Syn has a specific lifespan, directly determined by the chemical and physical properties of the monomer’s environment [4,90]. Any condition (e.g., membrane damage, overexpression) that diminishes the tetramer/monomer ratio, thereby increasing the level of disordered *α*Syn monomers, promotes aggregation, protofibril, and fibril formation, leading to cytotoxicity [4,90,91]. Understanding this, the relatively low abundance of tetrameric forms in the transgenic HR cultures suggested that these low tetramer/monomer ratios were conducive to aggregation-mediated cellular stress. Indeed, we observed that *α*Syn-A53T expression in HRs triggers a proteostatic stress syndrome similar to that observed in humans and animals.

While the proteostatic stress response was unsurprising, given the conservation of misfolded protein response mechanisms among eukaryotes, the accompanying change in iron homeostasis was less expected. There are essential differences in iron regulation between plants and animals. For example, the downregulation of ferritin, seen in both human and plant cells, has different regulatory components—translational in humans and transcriptional in plants, involving plant-specific regulatory proteins. The consistent downregulation of ferritin in response to toxic *α*Syn expression in both animal and plant cells indicates a crucial involvement of this iron homeostasis protein in the cellular stress induced by *α*Syn. It also implies a common mechanism in its downregulation, potentially independent of the differential transcriptional and translational controls.

The development of plant models for synucleinopathies holds clinical relevance as it expands our understanding of the disease and provides an additional tool for investigating its molecular pathophysiology. This may lead to the discovery of new diagnostic methods, biomarkers, or criteria that can enhance disease detection, monitoring, or risk assessment. Additionally, it offers a platform for identifying new pharmaceutical agents and evaluating the effectiveness of existing ones at a fraction of the costs associated with research in animal models and humans. Thus, our findings indicate that plants offer a triple advantage—serving as a model for investigating key aspects of pathological *α*Syn expression, a source of potential drug candidates for PD, and possibly a plant bioassay that can be used to screen for drugs that affect synucleinopathies. The credibility of using plant models to analyze common molecular denominators in *α*Syn pathologies is reinforced by the observed interaction between dopamine and iron in plant cells [92]. This interaction is pivotal, mirroring the *α*Syn/dopamine/iron interactions observed in synucleinopathies in human and animal systems [93,94]. The importance of the plant model in identifying potential PD drug candidates stems from the intrinsic ability of plants to generate a wide variety of species-specific secondary metabolites [27–30]. Notably, the *α*Syn-A53T expression in *L. cardinalis* resulted in a dosage-dependent increase in anthocyanin accumulation—a significant proof of concept, especially considering the demonstrated neuroprotective roles of specific anthocyanin species in a PD model [95]. Consequently, our results suggest the potential utilization of the expression of *α*Syn in medicinal plants recognized for their neuroprotective properties (such as the ones used in this study [31–36]) as a resource for discovering drugs that possibly affect the treatment and/or progression of PD.

## Supplementary Material

Kurepa et al., 2024 Supplementary materials

## Data Availability

The raw data supporting the conclusions of this article will be made available by the authors on request.
