# Supplementary material for: A Plant Model of α-Synucleinopathy: Expression of α-Synuclein A53T Variant in Hairy Root Cultures Leads to Proteostatic Stress and Dysregulation of Iron Metabolism
Source: Appl Biosci (Basel). Author manuscript; Available in PMC 2024 Jun 4. (PMC11149894; doi:10.3390/applbiosci3020016)
Supplement: Kurepa et al., 2024 Supplementary materials [file NIHMS1997253-supplement-Kurepa_et_al___2024_Supplementary_materials.docx]

Supplementary Materials

A Plant Model of α-Synucleinopathy: Expression of
α-Synuclein A53T Variant in Hairy Root Cultures Leads to
Proteostatic Stress and Dysregulation of Iron Metabolism

Jasmina Kurepa ^1^, Kristen A. Bruce ^2,†^, Greg A. Gerhardt ^3,4,5,6^ and Jan A. Smalle ^1,^*

^1^ Department of Plant and Soil Sciences, Martin-Gatton College of Agriculture Food and Environment,
Kentucky Tobacco Research & Development Center, University of Kentucky, Lexington, KY 40546, USA; jasmina.kurepa@uky.edu

^2^ Naprogenix, Inc.™, UK-AsTeCC, 145 Graham Avenue, Lexington, KY 40506-0286, USA;
kbruce42@yahoo.com

^3^ Brain Restoration Center, University of Kentucky, Lexington, KY 40536, USA; gregg@uky.edu

^4^ Department of Neurosurgery, University of Kentucky, Lexington, KY 40536, USA

^5^ Department of Neuroscience, University of Kentucky, Lexington, KY 40536, USA

^6^ Department of Neurology, University of Kentucky, Lexington, KY 40536, USA

***** Correspondence: jsmalle@uky.edu

^✝︎^ Current address: UES, Inc., 4401 Dayton-Xenia Rd., Dayton, OH 45432, USA.


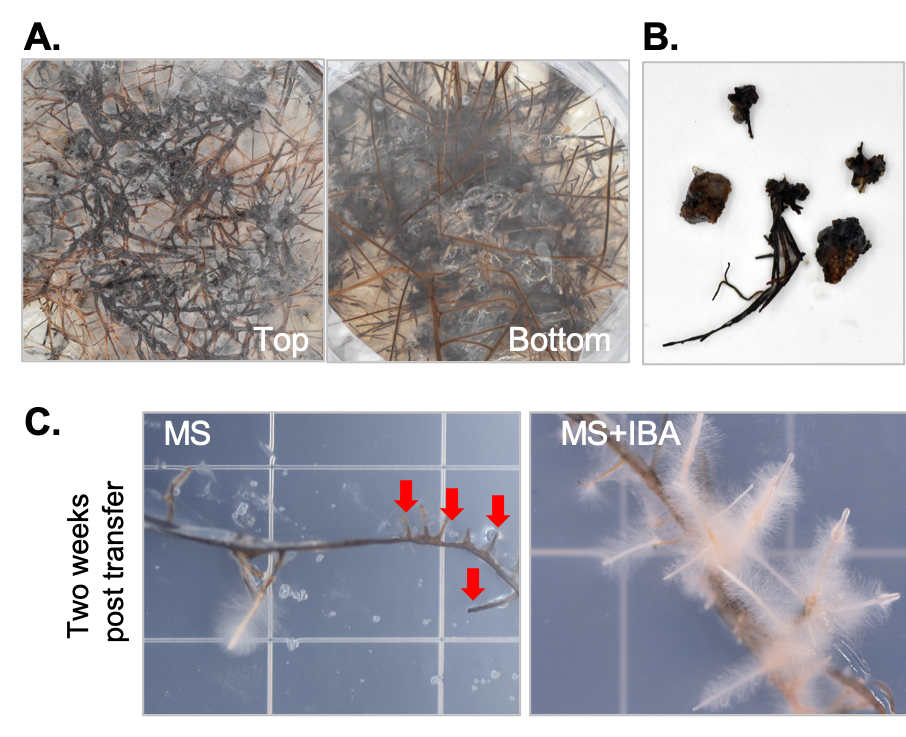


**Figure S1.** Growth characteristic of the *P. multiflorum* HR cultures. (**A**). The images illustrate a representative petri plate containing two-month-old cultures grown on MS media. Both top and bottom views are provided to highlight the HRs' limited ability to penetrate the media and their increased tendency to adhere to the air. (**B**). Calli formation observed in older *P. multiflorum* HR cultures. (**C**). The impact of auxin IBA (10 µM) on the growth of *P. multiflorum* HR cultures is demonstrated. As expected, IBA heightened the density and robustness of newly formed roots. However, the addition of IBA did not influence other growth characteristics (e.g., those illustrated on panels A and B) of the *P. multiflorum* HR cultures. Arrows indicate arrested (quiescent) lateral roots. It is important to note that plant hormones play a significant role in shaping the profile of secondary metabolites [28]. For example, auxins are known to be involved in the synthesis of various alkaloids, flavonoids, and phenolics. Thus, whereas cultivating *P. multifloru* HR on media without auxin limits the amount of analyzable tissue, it is still a preferred method of cultivation as it ensures that effects of the expression of αSyn remain the sole factor influencing the biosynthesis of secondary metabolites.
